# Managing normal pressure hydrocephalus in the developing countries; personalizing the treatment

**DOI:** 10.1007/s10143-025-03474-5

**Published:** 2025-04-10

**Authors:** Hussein Fathalla, Mohamed Adel Ghoneim, Amr el Katatny

**Affiliations:** Division of Neurosurgery, Cairo University Hospitals, Cairo, Egypt

**Keywords:** Normal pressure hydrocephalus, Developing countries, Gravitational shunts, Programmable shunts

## Abstract

The current recommended treatment for NPH is insertion of a programmable shunt. In developing countries however, this might sometimes not be feasible due to cost and logistical reasons. Patients in rural areas are required to travel hundreds of miles for frequent follow-up visits for reprogramming, rendering the treatment protocol troublesome and sometimes even not possible. Our approach was to treat these patients with standard gravitational shunts (MIETKE GAV) instead of programmable shunts and assess the outcome. 43 cases were treated by the authors from the period of 2018 to 2023. Only patients classified as probable NPH according to the INPH guidelines criteria were included. We used fixed pressure non-programmable gravitational shunts for all patients. Improvement was assessed using the Krauss method and only patients with 50% improvement or more were considered improved. There were 33 males and 10 females with a mean age was 74 years. Initially only 30 (69.7%) improved however, after a second surgery for 7 of the remaining 13 patients, the final number of improved patients were 35 (81.3%). Median follow up was 23 months. The use of fixed pressure gravitational shunts such as the GAV shunt, is a good second option for treating NPH patients in developing countries and rural communities. This approach provides a cheap, reliable, and practical solution for NPH patients living in rural areas, without the need for frequent follow-up visits. Although long-term follow-up is still needed, the success rate so far is satisfactory.

## Introduction

With a rapid aging society in the developing countries, the care for Normal Pressure Hydrocephalus (NPH) has become an important issue [1, 16]. The current recommendation for treating NPH according to the most recent guidelines is insertion of a programmable shunt device, preferably with an anti-siphon system [4, 12, 21, 23]. In developing countries however, this might sometimes not be feasible. Egypt for example, has a population of 110 million with a very complex social and demographic nature. While two thirds of them are currently living in cities with access to specialized neurosurgical centers, there remains a third (almost 30 million) living in rural areas, far away from specialized hospitals, and of low socioeconomic standard. An estimated 5 billion people from low- and middle-income countries currently has no access to basic surgical care [16]. Treating patients from these areas with a programmable shunt is very costly and requires travelling hundreds of miles for frequent follow-up visits for reprogramming, rendering the treatment protocol troublesome and sometimes even not possible. Moreover, many of the elderly population in rural areas around the world grew up in a time before worldwide educational reforms and are illiterate. Compliance to treatment and follow-up visits in these patients is a challenge.

It was therefore a pressing issue to find a practical personalized solution to our NPH patients living in rural areas. The solution must be cheap, reliable, and doesn’t require frequent follow-up visits. At the beginning we used to treat these patients with standard fixed-pressure valves (medium or low), which of course led to some complications of either over or under-drainage. It was only with the evolution of the gravitational shunts [9, 12], that we started implementing a different approach for this subset of patients.

These gravitational valves were developed to treat siphonage problems frequently seen with fixed differential pressure valves and have become the standard of care for CSF (cerebrospinal fluid) diversion in many European countries for pediatric and adult hydrocephalus [4, 9, 21]. While they are very efficient for treating many types of hydrocephalus, only one study reported its use specifically to treat NPH as a standalone device [14] given that the gold standard for NPH is now a programmable shunt and these easily available in the developed world. In our practice, we naturally opt for a programmable shunt + gravitational valve (MIETKE PROGAV with SA^®^, or MEDTRONIC STRATA VALVEⓇ with Delta chamber) for all our NPH patients as well, same as the standard of care and offer it as the first option. On many occasions however, there will be difficulties in providing it for our patients in rural areas. Our rationale was that if these gravitational valves can help with siphonage problems, then why not try them as a standalone solution for our patients living in remote and rural areas instead of the programmable valves.

Our approach was to treat these patients with standard gravitational shunt (MIETKE GAV^®^) instead of programmable shunts and assess the outcome. In this study we present our experience using these gravitational shunts to treat NPH patients living in remote and rural areas with excellent results.

## Methods

### Patients

**Fig. 1:**
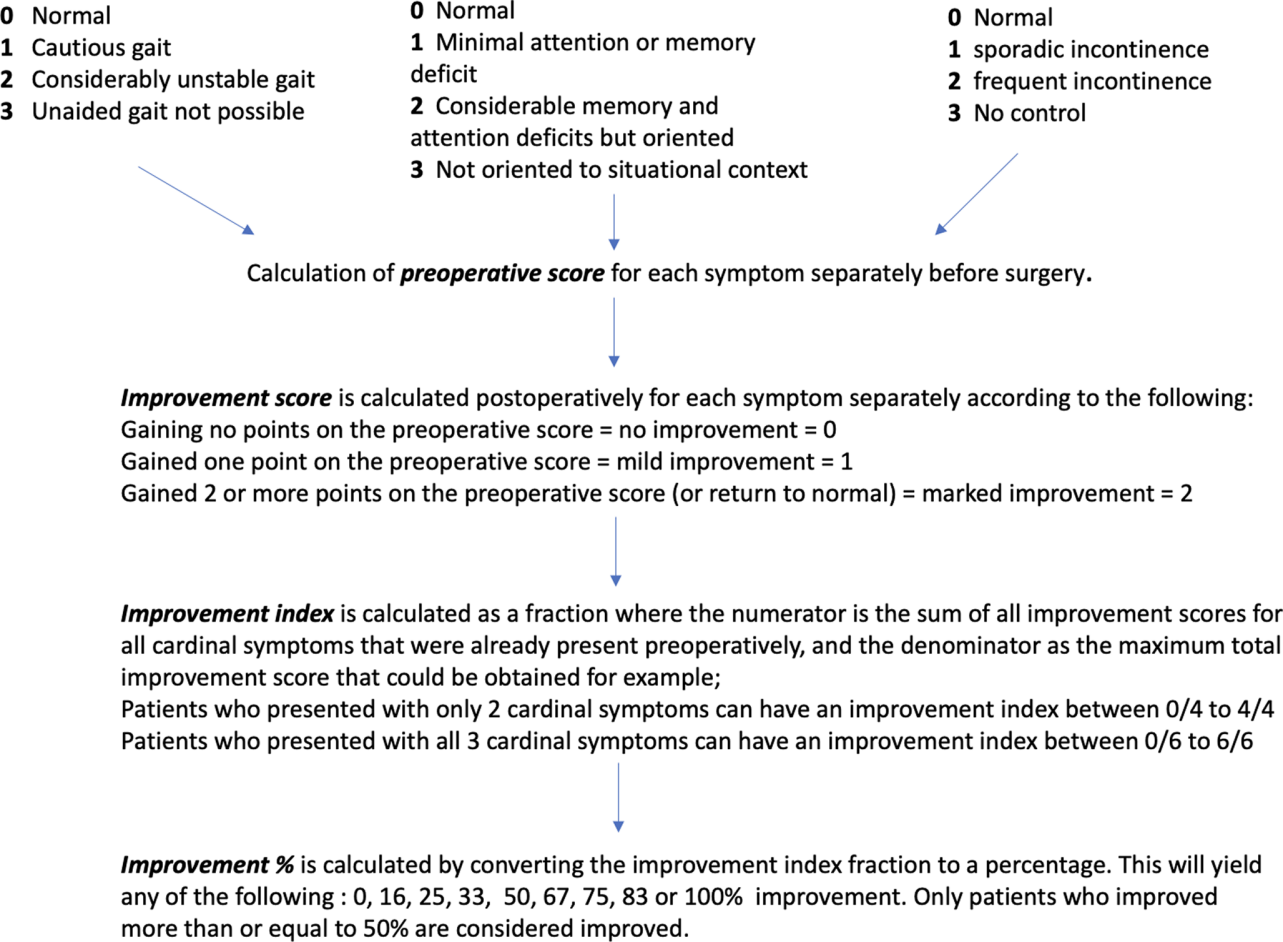
Calculation of preoperative score, improvement index and improvement percentage using the Kraus method [10]

43 cases were treated from the period of 2018 to 2023 and were included in this study. We only included patients from long-distance rural areas and low socioeconomic status who were not candidates for programmable shunts for obvious economic, social, and logistic reasons. Patients who are treated with programmable shunts are excluded from this study. All patients underwent a complete history, physical examination, and brain imaging with magnetic resonance imaging (MRI). Cognitive assessment was done using an Arabic version of the MOCA (Montreal cognitive assessment) test [17]. All patients were then classified as probable or possible INPH according to the INPH guidelines criteria [7, 19]. Patients who were classified as unlikely were not included in our study. Preoperative scores were calculated for each patient (Fig. 1). Although not a prerequisite for surgery, we offered surgery only after a positive CSF tap test thus ensuring that surgery will only be offered to patients with probable NPH. CSF flow resistance using the intrathecal test (IT) was not available in our practice due to financial constraints, although being a good prognostic indicator for shunt responsive [5, 8, 13, 15, 23].

### Shunts used

Our usual protocol for INPH patients is similar to the standard of care; inserting a programmable shunt with an antisiphon device (Medtronic strata valve with delta chamber or Mietke ProGav with a shunt assist device). In this study however, for patients in rural Egypt we used a Mietke GAV^®^ shunt, which is a fixed pressure non-programmable 5/30 gravitational shunt providing a range of opening pressure of 5 to 30 according to the patients’ position. This gravitational fixed pressure shunt was originally developed as a standalone valve to treat various CSF conditions or as an accessory to programmable valves (for NPH or any other type of hydrocephalus) while at the same time protecting patients from over drainage with the high opening pressure provided during the upright positions. It is worth noting that we preferred this device over the DSV (Dual switch valve) shunt as it provided a range of opening pressures (5 **to** 30) according to the degree of head elevation compared to the DSV that switches between either of only 2 pressures when upright or flat (5 **or** 30) [14].

### Improvement and follow up

Patients were followed up by a brain computed tomography CT scan on postoperative day 2 to confirm shunt tip position. Regular follow ups were done on day 14 for stitches removal and assessing the response to treatment, one month postoperative then every 6 months. Improvement was assessed using the Krauss method [11] (Fig. 1) and only patients with 50% improvement or more on their last follow up are considered improved.

### Statistical analysis

SPSS 20 software (SPSS Inc., Chicago, Illinois) was used for statistical analysis. Data were presented as mean and range for numerical variables (e.g. Age) and as frequency for categorical variables (sex, complications…etc.).

## Results

### Study population

The study population and demographics are listed in Table 1. There were 33 males (76.8%) and 10 (23.2%) females in our series. Mean age was 74 years. 35 patients (81.3%) displayed all three symptoms while 8 (18.6%) displayed only 2 symptoms, with gait disturbance being the symptom present in all patients. Preoperative symptoms and scores are listed in Table 2. Our median follow up was 23 months (range: 12–49).

**Table 1:** Demographics and patient characteristics

**SEX**	Number of patients (%)
Female	10 (23.2)
Male	33 (76.8)
**AGE**	
60–70	9 (20.9)
70–80	26 (60.4)
80–90	8 (18.6)
*Mean age ± SD*	74 ± 1.8
**Presenting symptoms**	
2	8 (18.6)
All 3	35 (81.3)
**Comorbidities**	
diabetes	25 (58.1)
Cardiovascular	31 (72)
Smoker	2 (51.1)
Obesity	12 (27.9)
**MEDIAN FOLLOW UP, MONTHS ± SD (range)**	23 ± 3.2 (12–49)

**Table 2:** Preoperative severity scores for all symptoms in all patients

Preoperative severity	Gait	Dementia	Incontinence
0	0 (0%)	6 (13.9%)	2 (4.6%)
1	5 (11.6%)	17 (39.5%)	20 (46.5%)
2	27 (62.8%)	12 (27.9%)	14 (32.6%)
3	11 (25.6%)	8 (18.6%)	8 (18.6%)

### Complications

**Fig. 2:**
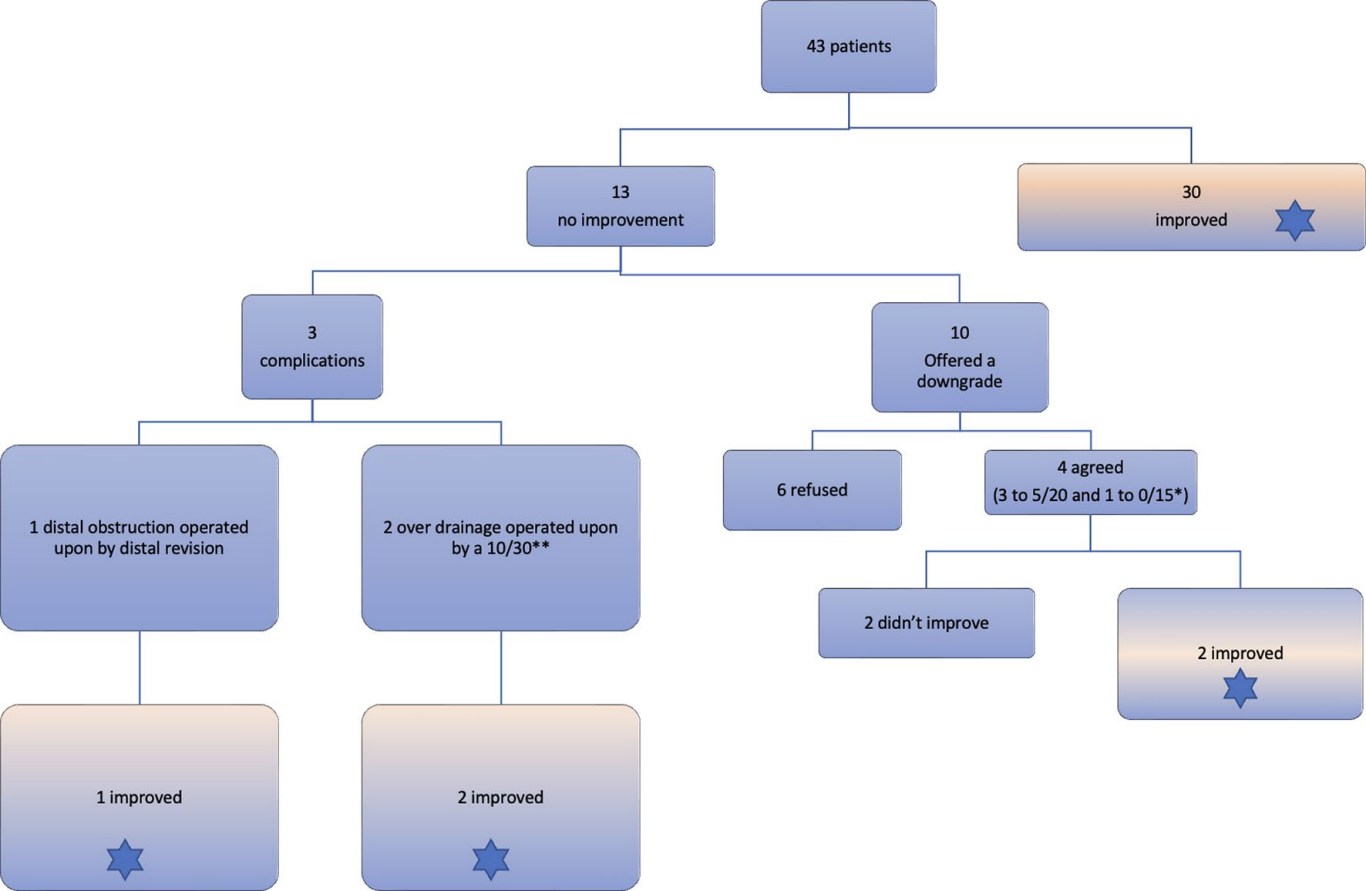
Flowchart for our patients. *Heart failure patient who couldn’t lie flat, so we chose a lower pressure without concern of overdrainage. ** one of them was a bedridden patient. *Blue star*: Improved patients

We had 3 complications in 3 patients (7%); one patient suffered a distal catheter obstruction who underwent distal revision, and 2 patients suffered from over drainage and presented with severe headache and subdural collections. All 3 of them required a second surgery (Fig. 2**)**.

### Improvement

A flow chart of the sequence of events for all patients is presented in Fig. 2. Initially, 30 (69.7%) of the 43 patients were classified as improved (improvement more than 50% using Kraus improvement index). Of the 13 patients who didn’t initially improve, 10 were offered another surgery to downgrade to a lower pressure setting shunt but only 4 agreed (3 patients to GAV 5/20 and 1 patient to 0/15). 2 of them improved after the downgrade. After managing these 4 patients together with the patients who suffered the complications listed above, the total number of patients who improved on final follow up were 35(81.3%) out of the 43. Table 3 lists the improvement indexes and percentages for all our series.

**Table 3:** Final improvement index and percentage after managing all complications. 35 patients (81.3%) improved on final follow up

Improvement index (%)	Number of patients	Percentage
0/4 (0%)	1	2.3
1/4 (25%)	1	2.3
***2/4 (50%)***	***2***	***4.6***
***3/4 (75%)***	***3***	***6.9***
***4/4 (100%)***	***1***	***2.3***
0/6 (0%)	1	2.3
1/6 (16%)	3	6.9
2/6 (33%)	2	4.6
***3/6 (50%)***	***7***	***16.2***
***4/6 (67%)***	***10***	***23.2***
***5/6 (83%)***	***7***	***16.2***
***6/6 (100%)***	***5***	***11.6***
Total improvement	*35 / 43*	*81.3*

## Discussion

The current recommendation for treating NPH according to the most recent guidelines is insertion of a programmable shunt device, preferably with an anti-siphon system. In developing countries like Egypt, there is a large rural population who don’t have access to expensive programmable shunts and will not comply with follow-up instructions. Our aim was to find another solution for this specific population.

Initially we either used fixed pressure valves with low or medium pressure for this subset of patients, but the complications rates were difficult to ignore for both options. The quoted rates in the literature for over drainage symptoms are 17–50%, and for subdural haemorrhage are (~ 13%) [20, 24]. These rates will probably increase more with low pressure shunts. Medium pressure shunts will also have siphonage problems like all shunts but in addition can also fail to relieve the symptoms because of inadequate drainage as compared to lower pressure shunts [6]. With the emergence of gravitational shunts, it was obvious that they could solve this dilemma. In addition to being cheap, these devices are easy to insert, requires no postoperative programming, provide good drainage in the supine position due to their low opening pressure while maintaining a high opening pressure in the upright position to protect against siphonage and over drainage.

### Postoperative assessment methods

All improvements were assessed using the Krauss measure [11] to generate an improvement percentage% (Fig. 1). In our study, only patients with 50% improvement or more were considered improved. In our opinion, any value less than that simply means that the patient only obtained a very mild improvement in only one of his presenting symptoms so couldn’t be classified as true improvement. We believe this is a very realistic method as some papers would rely more on subjective measures and consider minor improvements as a true improvement in their statistics.

### Results and complications

In this study, ~ 70% of patients improved on long term follow up without complications or sequalae. This is somewhat lower but very close to the rate of improvement in recent literature [3, 10, 15, 22, 23]. While this treatment method is not optimal, we believe it provides relatively good results for this subset of patients compared to no treatment. Moreover, it should be highlighted that our improvement criteria (minimum 50% improvement) could have contributed to this lower success rate. After management of some of the non-improved patients and/or complications with a second surgery however, our success rate rose to 81.3%. on the final follow up.

10 patients didn’t improve and only 4 of them agreed to a second surgery, where we opted for a downgrade to a 5/20 valve for 3 of them and 0/15 to one. While the 0/15 patient improved, only one of the 3 who had the downgrade to 5/20 improved. This raises a question of whether we should have inserted a 0/15 also to these 3 patients however, we continue to be reluctant to do so from fear of over drainage. Our only patient who had the 0/15 had a heart condition and couldn’t ever lie down flat, so we didn’t have this concern with him. The scenarios with these 10 non-improved patients are where the superiority of the programmable shunts is evident.

We had only 3 complications in 3 patients (7%); 1 distal obstruction who underwent distal revision, and 2 patients suffering from over drainage and presenting with subdural collections. One of them required a subdural evacuation surgery. Both had their shunt upgraded to 10/30. All 3 improved after managing their complications and continued to do well (Fig. 2).

### Choice of pressure and individualization for special cases

There are multiple pressure ranges available in the market. Our initial choice was a GAV 5/30 shunt in all patients. This is in-line with the manufacturer’s recommendation and was deemed appropriate by the authors. Theoretically, one should choose a device with a lower number for the upright position in shorter patients less than 160 cm (5/20) however, we didn’t have any patients in this height range.

While the choice of the 5/30 shunt proved successful, we had 2 patients who taught us a great deal when choosing the pressure of the GAV shunt initially. The first lesson was learned from one of our two patients who suffered from an over drainage. He was a bedridden patient due to an old spine condition causing severe chronic pain and depression. This patient suffered from a subdural hemorrhage causing severe headache and slight motor weakness that required surgical decompression and a shunt upgrade. The reason we speculated is that he was always under the effect of an opening pressure around or slightly higher than 5 cmH2O as he was rarely in an upright position making it easy to over drain, although a counter argument is that being rarely in an upright position would theoretically mean more protection from siphonage and overdrainage which may contradict our explanation. Regardless, we immediately changed the shunt to a 10/30 after evacuation of the subdural collection. He improved immediately afterwards and continued to do well. The second patient had the opposite problem; he was a patient with severe heart failure and could not tolerate a head down position. He would always stay in an upright or semi sitting position even during his sleep. This patient had not improved and continued to deteriorate from his NPH due to underdrainage as he was always under the effect of the shunt in the upright position with a high opening pressure around the 30 cmH2O. While theoretically this could cause more siphonage and drainage however, an easier explanation is to imagine him as an NPH patient who has a fixed differential high pressure pressure valve (~ 15–30) making it difficult to improve. In this patient we substituted the GAV unit for a 0/15 SA unit. The patient improved and continued to do well on his last follow-up. It is to be noted that this is not always a problem as many heart failure patients will be able to sleep in a left decubitus position and thus might still do well with the 5/30 shunt. Moreover, these patients who sleep in a lateral decubitus position will be at risk of over-drainage if you decide to change the shunt to a 0/15 in case they don’t improve initially with the 5/30 shunt, so the 0/15 shunt should not be done routinely to patients who doesn’t respond. Care should be taken when assessing patients who has not improved with a 5/30 shunt before you decide the next step. Those 2 complications made us aware of the variables that needed to be assessed preoperatively before choosing the appropriate pressure. A 5/30 shunt is an excellent pressure setting for most patients however, patients who are bed-ridden or who usually need to be in an upright position might require other options. These are situations where the superiority of programmable valves is evident.

### Limitations

In addition to the retrospective nature of this study, several issues need to be considered. NPH is a progressive disease [2] and if deterioration occurs, the patient will require a lower pressure setting. Since many of our patients do not have long term follow-ups due to distance and socioeconomic status (our max follow-up is 4 years), we don’t not know their long-term outcomes. It is possible that some of them will decline later and need another surgery however, our minimum follow up is one year and all our improved patients remained stable at their last follow-up. One year follow-up is generally considered a good cutoff time to determine improvement as deterioration past this mark is sometimes attributed to comorbidities and advancing age [10]. This makes us optimistic that most of our patients them will continue to do well.

Another important limitation is that our selection criteria were more stringent than many other studies. We only included patients with probable NPH and not for the unlikely category. These selected patients were only offered surgery in case they had a positive tap test. It is agreed that some patients in the possible or unlikely category and/or patients with negative tap tests could still improve with continuous lumbar drainage or infusion tests, and thus could be offered surgey [13]. Patients in the unlikely or possible category however would lower the success rates if they were included in this series. This selection bias might have led to somewhat higher success rates in our series. We have tried to offset this effect by using vigorous improvement criteria (at least 50% improvement) which could have in turn lowered our success rate.

Finally, we should emphasize that although our management might have offered a solution for these patients living in rural areas, other countries or centers dealing with patients in rural areas with low socioeconomic conditions might find this line of treatment not cost-effective. A good example is a study by Reis and Pinto et al. [18] that reported good results in brazil using fixed pressure valves for NPH. They noted that although programmable valves are much more expensive than fixed pressure valves, the total cost of treatment using fixed pressure valves was much higher after accounting for a rate of 25% re-operations and complications with fixed pressure valves. In our study we had a re-operation rate of 16% and that could have been much higher if all our non-improved patients agreed to a second surgery. Nevertheless, perhaps because there are huge variations in the cost of surgeries and medical services between regions and countries, in our experience the cost was still much lower than if we have used a programmable valve. It is thus advisable for each center to do a feasibility and cost analysis study before attempting this line of treatment.

## Conclusion

An estimated 5 billion people mostly in low- and middle-income countries have no access to basic surgical care. Programmable shunts are not feasible in this specific subset of patients due to economic, social, and logistic constraints. The use of fixed pressure gravitational shunts such as the GAV shunt, is a good second option for treating NPH patients in developing countries and rural communities. This approach provides a cheap, reliable, and practical solution for NPH patients living in rural areas, without the need for frequent follow-up visits. Although long-term follow-up is still needed, the success rate so far is satisfactory.

## Data Availability

No datasets were generated or analysed during the current study.

## Abbreviations

NPH: Normal pressure hydrocephalus
CSF: Cerebrospinal fluid
MRI: Magnetic resonance imaging
MOCA: Montreal cognitive assessment test
DSV: Dual switch valve
CT: Computed tomography
